# Is omission of free text records a possible source of data loss and bias in Clinical Practice Research Datalink studies? A case–control study

**DOI:** 10.1136/bmjopen-2016-011664

**Published:** 2016-05-13

**Authors:** Sarah J Price, Sal A Stapley, Elizabeth Shephard, Kevin Barraclough, William T Hamilton

**Affiliations:** 1 Medical School, University of Exeter, College House, Exeter, UK; 2 Hoyland House, Painswick, UK

**Keywords:** PRIMARY CARE, STATISTICS & RESEARCH METHODS, ONCOLOGY

## Abstract

**Objectives:**

To estimate data loss and bias in studies of Clinical Practice Research Datalink (CPRD) data that restrict analyses to Read codes, omitting anything recorded as text.

**Design:**

Matched case–control study.

**Setting:**

Patients contributing data to the CPRD.

**Participants:**

4915 bladder and 3635 pancreatic, cancer cases diagnosed between 1 January 2000 and 31 December 2009, matched on age, sex and general practitioner practice to up to 5 controls (bladder: n=21 718; pancreas: n=16 459). The analysis period was the year before cancer diagnosis.

**Primary and secondary outcome measures:**

Frequency of haematuria, jaundice and abdominal pain, grouped by recording style: Read code or text-only (ie, hidden text). The association between recording style and case–control status (χ^2^ test). For each feature, the odds ratio (OR; conditional logistic regression) and positive predictive value (PPV; Bayes’ theorem) for cancer, before and after addition of hidden text records.

**Results:**

Of the 20 958 total records of the features, 7951 (38%) were recorded in hidden text. Hidden text recording was more strongly associated with controls than with cases for haematuria (140/336=42% vs 556/3147=18%) in bladder cancer (χ^2^ test, p<0.001), and for jaundice (21/31=67% vs 463/1565=30%, p<0.0001) and abdominal pain (323/1126=29% vs 397/1789=22%, p<0.001) in pancreatic cancer. Adding hidden text records corrected PPVs of haematuria for bladder cancer from 4.0% (95% CI 3.5% to 4.6%) to 2.9% (2.6% to 3.2%), and of jaundice for pancreatic cancer from 12.8% (7.3% to 21.6%) to 6.3% (4.5% to 8.7%). Adding hidden text records did not alter the PPV of abdominal pain for bladder (codes: 0.14%, 0.13% to 0.16% vs codes plus hidden text: 0.14%, 0.13% to 0.15%) or pancreatic (0.23%, 0.21% to 0.25% vs 0.21%, 0.20% to 0.22%) cancer.

**Conclusions:**

Omission of text records from CPRD studies introduces bias that inflates outcome measures for recognised alarm symptoms. This potentially reinforces clinicians’ views of the known importance of these symptoms, marginalising the significance of ‘low-risk but not no-risk’ symptoms.

**Strengths and limitations of this study**

- Set in the Clinical Practice Research Datalink, in which electronic medical records are recorded prospectively, eliminating recall bias.
- Large study size.
- Comprehensive identification of coded records of cancer symptoms.
- Search criteria for identifying text records of cancer symptoms will have missed US spellings and spelling errors.
- Study did not identify whether text records arose through the recording practices of a small number of general practitioner practices.

## Introduction

The Clinical Practice Research Datalink (CPRD) is a UK-based research service that maintains a database of anonymised copies of medical records collected by general practitioners (GPs) as part of everyday clinical care. The database is the largest of its kind and a representative sample of the UK population. Though created for pharmacological research, the database is now used extensively in epidemiological studies.^1^ ^2^ Quality standards for the CPRD—devised for pharmacoepidemiology research—have not been amended to accommodate areas such as symptom-based research.

One potential, but neglected, concern for those using CPRD data arises from the way clinical events are recorded in the GP surgery. Most GP practices that contribute data to the CPRD use ViSion software (ViSion INPS, London, UK), in which GPs must choose a Read code to begin a record. Read codes have been used in the National Health Service to record patient findings since 1985, and there are now over 96 000 of them organised in a hierarchical classification system. Once a Read code has been chosen, a comments box opens in which GPs can type freely and are not limited to elaborating on the code. While codes are fully and routinely available to researchers, text records are not. Furthermore, a moratorium on the collection of text data by the CPRD was introduced in 2013. Text recorded before 2013 can be accessed by researchers, but this is rarely performed because the methods are complex, expensive and limited. Therefore, to be detected by researchers, clinical information in the medical record must be recorded using a Read code. Consequently, the omission of text records may introduce a ‘detection’ bias, because researchers are oblivious to clinical events that are only ever recorded as an inaccessible text comment (henceforth called ‘hidden text’).^3^

In a previous CPRD case–control study, we identified that, of 312 patients with non-visible haematuria, records of this fact were made solely in hidden text for 219 (59%). The proportional loss of records in the hidden text was similar in cases and controls. Therefore, the effect of the detection bias was limited to underestimation of the frequency of non-visible haematuria.^4^

This paper further investigates the potential consequences for research of having two recording methods—codes and hidden text. Specifically, we sought evidence for the disproportionate use of hidden text to record symptoms/signs presented by cases compared with controls, as this will result in biased estimates of outcome measures such as ORs and positive predictive values (PPVs). This is important, because the outputs of such research are used as evidence to underpin national guidelines; for example, estimates of cancer risk in symptomatic patients presenting to primary care.^5^ ^6^

## Method

This study extended two CPRD studies conducted by our group that identified the features of bladder and pancreatic cancer in primary care.^5^ ^6^ This was deliberate, in order to investigate whether detection bias affects the many cancer diagnostic studies conducted since 2000, all restricting their analysis to coded CPRD data.^5–13^

### Setting, patients and period of study

CPRD data were used in a matched case–control design. Cases (≥40 years old) had a bladder/pancreatic cancer-specific code recorded between 1 January 2000 and 31 December 2009 inclusive. Each case was matched with up to five controls on age, sex and GP practice. The diagnosis date for each case, taken as their earliest recorded cancer-specific code, determined the end point of the 1-year analysis period for each case–control set. Full inclusion and exclusion criteria for participants are available in online supplementary appendix 1.

10.1136/bmjopen-2016-011664.supp1 Supplementary appendix 1

### Variables

We examined whether omitting text records from analysis results in the underestimation of the frequency of three features of cancer. These were chosen as being representative of ‘alarm’ or of ‘low-risk but not no-risk’ features of cancer; namely, visible haematuria (an alarm symptom for bladder cancer), jaundice (an alarm feature of pancreatic cancer) and abdominal pain (a low-risk symptom common to both cancers).^5^ ^6^

#### Identifying coded records of visible haematuria, jaundice or abdominal pain

The medical records of all participants were searched for Read codes for visible haematuria, jaundice and abdominal pain.

The original bladder cancer study's list of codes for visible haematuria was reused.^5^ Patients whose medical record contained none of the codes were assumed not to have experienced this symptom. This process was repeated using the jaundice code list created in the pancreatic cancer study.^6^

For abdominal pain, which is common to both cancers, the original studies had used slightly different code lists. Codes for indigestion and dyspepsia were included in the pancreatic, but not the bladder, cancer study. For the study reported here, a unified abdominal pain code list was constructed (thus, numbers for abdominal pain differ slightly from those originally reported).

Full code lists for visible haematuria, jaundice and abdominal pain are in online supplementary appendix 2.

10.1136/bmjopen-2016-011664.supp2 Supplementary appendix 2

#### Identifying text records of visible haematuria, jaundice or abdominal pain

CPRD were asked to search the hidden text of all participants for the following terms (optimised to catch variations in capitalisation):

- Abdominal pain: ‘bdominal pai’, ‘bdo pai’, ‘ain in abdo’, ‘pigastric pai’;
- Jaundice: ‘aundice’, ‘cterus’, ‘cteric’;
- Haematuria: ‘aematuria’, ‘lood in urine’.

CPRD anonymised each extract and provided text strings of the search term plus three words on either side to aid interpretation.

#### Generating variables from hidden text extracts

Hidden text extracts were converted to binary variables (symptom present/absent) using an algorithm run in Stata. The default classification was ‘symptom present’, and then extracts were classified as ‘symptom absent’ if they contained descriptors suggesting symptom negation (eg, ‘no visible haematuria’), or as ‘uncertain’ if they contained words suggesting that the meaning may be unclear (eg, ‘if any’). The classification of extracts flagged as ‘uncertain’ or as ‘symptom present’ was subsequently reviewed manually in consultation with WTH and KB, both practising GPs. If an extract's meaning could not be agreed, or it was confirmed that the extract was uninterpretable, the symptom was deemed to be absent. Summary information regarding the classification of all hidden text extracts is provided in online supplementary appendix 3.

10.1136/bmjopen-2016-011664.supp3 Supplementary appendix 3

The validity of the classification process was assessed by comparing its output with that of a gold standard. The gold standard was created using the panel of consensus method, in which WTH and KB independently rated a random sample of 84 text extracts about visible haematuria.^14^ The level of agreement between the algorithm and the gold standard was high (chance-corrected weighted κ =0.9, 95% CI 0.7 to 1.1). Full details of gold standard construction are in online supplementary appendix 3.

Doctors may make spelling and typographical errors when typing text records. Therefore, the random sample of text extracts used to create the gold standard was proofread and checked, and the number of American and misspellings reported.

#### Identifying text-only (hidden text) recording

For each patient, date matching of coded and text records identified where the GP had recorded visible haematuria solely in the text, that is, in hidden text. Binary variables were created to identify occurrences of visible haematuria that were overlooked in the original studies because GPs never used a code to record the event. The process was repeated for jaundice and abdominal pain records.

Each patient was then categorised by a binary variable denoting the style used to record their first five attendances for haematuria—subsequent attendances were omitted as they were so infrequent. ‘Coded’ was assigned if any record was made in coded form; conversely, ‘hidden text’ was designated only when all instances were noted solely in the text. This binary variable identified which patients with at least one episode of visible haematuria were overlooked in the original studies because the GP had never used a code to record the event. The process was repeated for jaundice and abdominal pain records.

### Analysis

Event-level analysis quantified how many visible haematuria records are lost to analysis by identifying the proportion of total records (coded plus hidden text) made in hidden text. Patient-level analysis quantified how many patients with visible haematuria are lost to analysis because their history of the symptom is always recorded in hidden text. Event-level and patient-level analyses were repeated for jaundice and abdominal pain. Associations between recording style and case–control status were also examined at the patient level for all features in both cancer data sets using the χ^2^ test (threshold p value <0.05).

The strength of association between a feature and cancer was assessed using univariable conditional logistic regression. ORs (95% CI) are reported before and after addition of previously hidden text records. The chance of cancer in patients presenting with a feature was estimated by the PPV (Bayes’ theorem), before and after addition of previously hidden text records.^15^

All analyses were conducted using Stata (V.13, StatCorp, College Station, Texas, USA).

## Results

### Patient characteristics

The participants’ characteristics are given in table 1 (see original studies for further details).^5^ ^6^

**Table 1:** Patient characteristics

Cancer site	Case–control	Number (%) in age group:	
		<60 years	≥60 years
Bladder	Cases (n=4915)	557 (11)	4358 (89)
	Controls (n=21 718)	2270 (10)	19 448 (90)
Pancreas	Cases (n=3635)	561 (15)	3074 (85)
	Controls (n=16 459)	2332 (14)	14 127 (86)

### Processing of text extracts

The CPRD provided 13 853 text extracts containing any of the search terms listed above in the Methods section. Details of their classification as ‘symptom present’, ‘symptom absent’ and ‘unclear’ are given in online supplementary appendix 3 tables 6 and 7.

The random sample of extracts used to create the gold standard contained 762 words, of which five were misspelt, representing an error rate of 0.7%. No instances of US instead of UK spelling—for example, ‘anemia rather than ‘anaemia’—were found.

### Quantity of data in the hidden text

**Figure 1:**
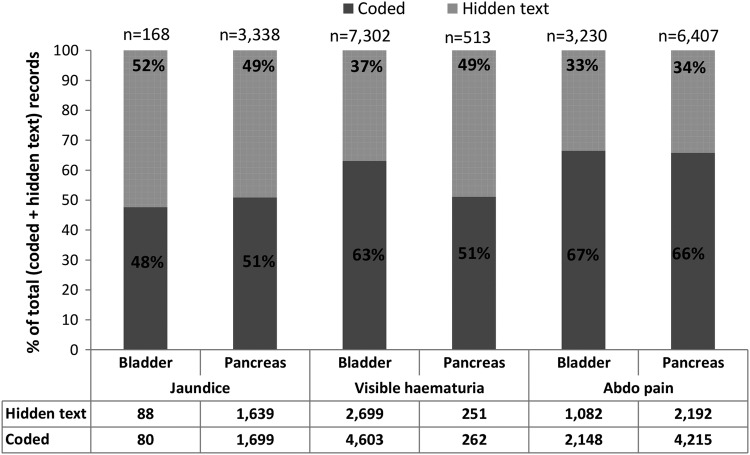
Event-level analysis: numbers of visible haematuria, jaundice and abdominal pain records in bladder and pancreatic cancer data set, grouped by recording method (dark grey: coded; light grey: solely in the text, ie, hidden text). Numbers of records are tabulated beneath the graphs, and the total number of records (codes+hidden text) is marked at the top of each bar. The percentage of records attributed to each recording style is marked on the bars.

At the event level, considerable numbers of visible haematuria, jaundice and abdominal pain records were made solely in the hidden text in both cancer data sets (figure 1) (see also online supplementary appendix 3 tables 6 and 7).

The proportion of records overlooked in the original studies because they were in the hidden text varied with the symptom. For visible haematuria, the proportion of total records made in hidden text was greater in the pancreatic (251/513=49%) than in the bladder (2699/7302=37%) cancer data set (figure 1). In both cancer data sets, approximately half of all jaundice records and one-third of all abdominal pain records were made solely in hidden text (figure 1). The Read code paired with hidden text records tended to be administrative (eg, telephone call), indicating the context of the consultation, or for another clinical problem (eg, another symptom or a diagnosis).

**Figure 2:**
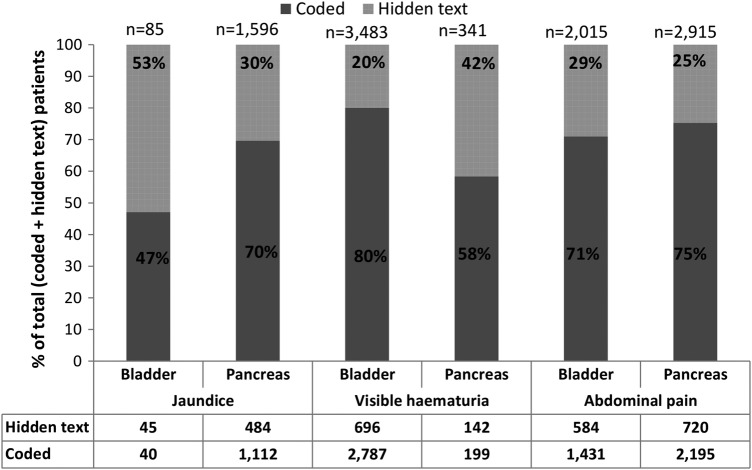
Patient-level analysis: numbers of patients with visible haematuria, with jaundice and with abdominal pain in bladder and pancreatic cancer data sets, grouped by recording method (dark grey: coded; light grey: solely in the text, ie, hidden text). Numbers of patients are tabulated beneath the graphs, and the total number of patients (codes+hidden text) is marked at the top of each bar. The percentage of patients attributed to each recording style is marked on the bars. Note: at the patient level, patients were categorised as having a hidden text recording style if all their attendances were documented solely in the text.

The patient-level data suggested that hidden text was used frequently and consistently, such that the original studies overlooked up to one-third of patients with these features. For abdominal pain, the proportion of patients lost to analysis was similar in both cancer data sets (bladder: 584/2015=29%; pancreas: 720/2915=25%) (figure 2). For alarm features, use of hidden text varied with the feature's strength of association with the cancer subsequently diagnosed in the case. For visible haematuria, only 696 of 3483 patients (20%) with this symptom in the bladder cancer data set were overlooked in the original studies; however, this proportion was doubled (142/341=42%) in the pancreatic cancer data set. A similar pattern was observed for jaundice recording.

### Associations between case–control status and recording style of alarm symptoms

**Figure 3:**
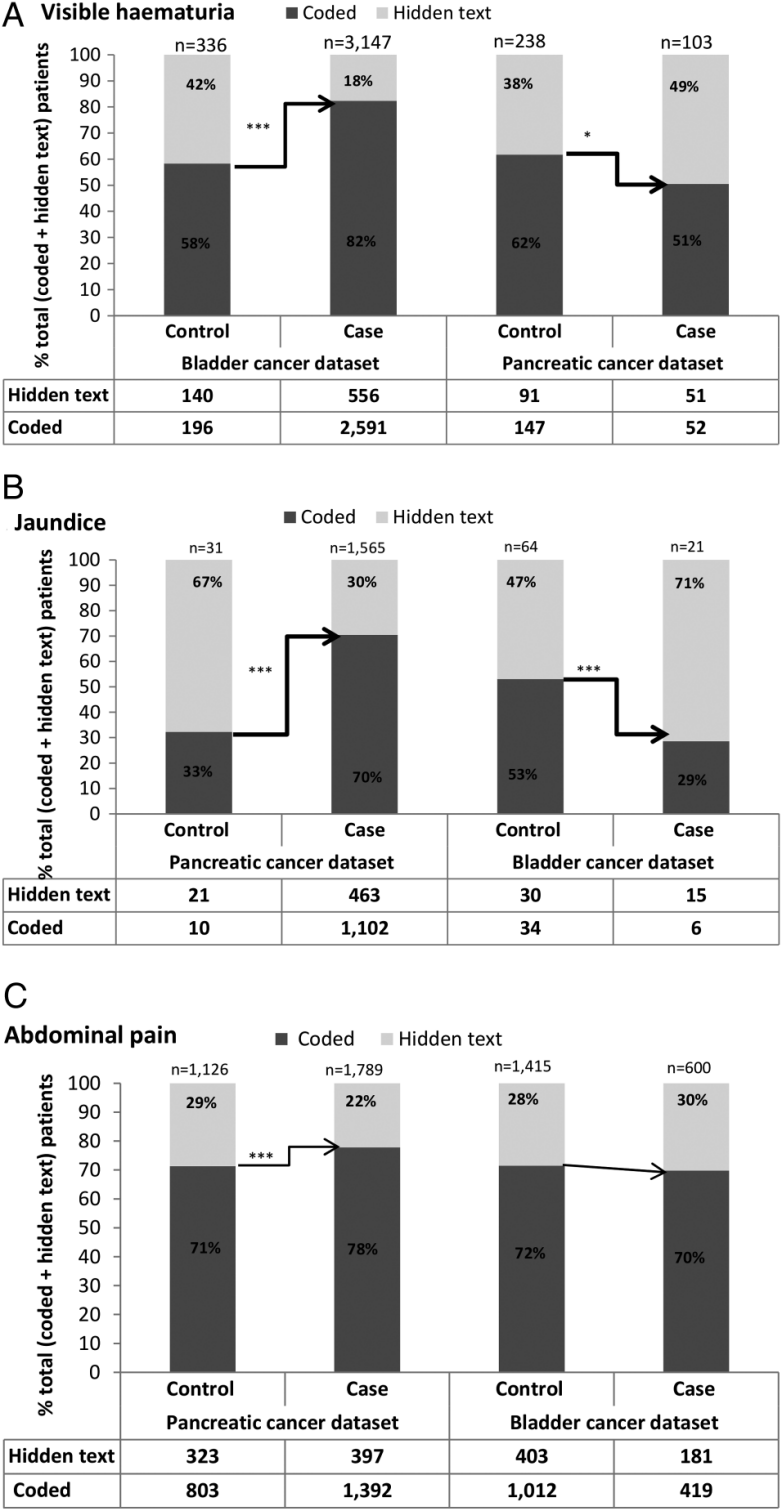
(A–C) Patient-level analysis. (A) Number of patients with visible haematuria in the bladder (left) and pancreatic (right) cancer data sets, grouped by recording method (dark grey: coded; light grey: solely in the text, ie, hidden text) and patient status (case or control). (B) Number of patients with jaundice in the pancreatic (left) and bladder (right) cancer data sets grouped by recording method and patient status. (C) Number of patients with abdominal pain in the pancreatic (left) and bladder (right) cancer data sets, grouped by recording method and patient status. Numbers of patients are tabulated beneath the graphs, and the total number of patients (codes+hidden text) is marked at the top of each bar. The percentage of patients attributed to each recording style is marked on the bars. Note: ***=p<0.001; *0.05>p>0.01.

For visible haematuria in the bladder cancer data set, there was a strong association between case–control status and recording style. The extent of patient loss in the hidden text was greater for controls (140/336=42%) than for cases (556/3147=18%) (χ^2^ test, p<0.001, figure 3A, left). However, in the pancreatic cancer data set, the pattern was reversed in that there was greater loss of cases with this symptom (51/103=49%) than controls (91/238=38%) (p<0.05, figure 3A, right). This reversal of bias was mainly driven by a large increase in the proportion of cases rendered as hidden text, the proportional loss of controls remaining relatively unchanged at ∼40%.

A similar pattern was observed for recording of jaundice which is strongly associated with pancreatic cancer. The extent of patient loss in the hidden text was greater for controls (21/31=67%) than for cases (463/1565=30%) in the pancreatic cancer data set (p<0.001) (figure 3B, left). As above, the bias was reversed in the unconnected cancer. In the bladder cancer data set, the loss of controls with jaundice (30/64=47%) was smaller than the loss of cases (15/21=71%) (p<0.001, figure 3B, right).

### Associations between case–control status and recording style of ‘low-risk but not no-risk’ symptoms

The association between case–control status and recording style is reported for abdominal pain in figure 3C. In the pancreatic cancer data set, hidden text recording affected a greater proportion of controls compared with cases (χ^2^ test, p<0.001), although the size of the difference was relatively small (controls: 323/1126=29% vs cases: 397/1789=22%). By contrast, in the bladder cancer data set, the records for ∼30% of controls and cases (p=0.4) were lost in hidden text (right-hand bars, figure 3C), mirroring the pattern seen in pancreatic cancer controls.

### Effect on ORs and PPVs

Minimal or no overlap of the CIs suggests that PPV values for visible haematuria and jaundice in their associated cancers were reduced by addition of hidden text records (table 2). The OR for bladder cancer in patients presenting with a single episode of visible haematuria tended to be decreased by addition of hidden text records, whereas the OR for jaundice was unaffected. Risk estimates for abdominal pain were not altered by addition of hidden text records (table 2).

**Table 2:** Odds ratios (OR) and positive predictive values (PPV) for cancer in symptomatic patients.

Cancer site	Symptom	OR (95% CI)		PPV (%) (95% CI)	
		Codes	Total	Codes	Total
Bladder	Visible haematuria	100 (78 to 129)	76 (61 to 95)	4.0 (3.5 to 4.6)	2.9 (2.6 to 3.2)
	Abdominal pain	1.9 (1.7 to 2.2)	2.1 (1.9 to 2.3)	0.14 (0.13 to 0.16)	0.14 (0.13 to 0.15)
Pancreas	Jaundice	713 (339 to 1499)	640 (354 to 1159)	12.8 (7.3 to 21.6)	6.3 (4.5 to 8.7)
	Abdominal pain	14.0 (12.6 to 15.6)	15.4 (13.9 to 17.0)	0.23 (0.21 to 0.25)	0.21 (0.20 to 0.22)

OR and PPV values were estimated first using codes (Codes) solely, and then using codes plus hidden text records (Total).

The OR was estimated using univariable conditional logistic regression. The PPV was estimated using Bayes’ theorem assuming prior odds of 0.0006 for bladder and 0.0003 for pancreatic cancer based on 2008 UK national incidence data.

## Discussion

### Principal findings

This study examined a neglected aspect of using CPRD data in symptom-based research; namely, loss of data due to recording style. For large numbers of patients, GPs record occurrences of visible haematuria, jaundice or abdominal pain solely in hidden text. Consequently, restricting analysis to codes underestimates symptom frequency, in patients with and in those without cancer. This confirms and extends our previous findings relating to non-visible haematuria.^4^

The major new finding of this study is that, for visible haematuria and jaundice, recording style choices lead to differential loss of information in hidden text between cases and controls. This suggests that most case–control studies of CPRD data analysing alarm symptoms are likely to be vulnerable to similar detection biases, because it is standard practice to restrict analysis to codes. When identifying patients with alarm symptoms, the bias works in favour of those subsequently diagnosed with cancers specific to the alarm symptom, and against those who either do not have, or are diagnosed with an unconnected, cancer. The detection bias thus artificially inflates PPV values for alarm symptoms in their associated cancers.

For abdominal pain, which is more strongly associated with pancreatic than with bladder cancer,^5^ ^6^ a minor detection bias favours identification of cases with abdominal pain, but only in the pancreatic cancer data set. Risk estimates are unaffected.

The results suggest that GPs are making strong clinical judgements about the probable significance of symptoms—preferentially coding clinical features they consider significant to a diagnosis, while tending to use hidden text to record those that they think are not. In this way, GPs are filtering electronic medical records available to researchers in favour of established associations between symptoms and diagnoses.

### Strengths and limitations

The main strengths of this study are its large size and that most clinical records were made at the time of the consultation, limiting if not eliminating recall bias. This study, like all those based on medical records, was limited by reliance on symptom reporting by patients and subsequent recording by GPs.

The study of hidden text records brought additional limitations. First, the search criteria will have missed US spellings (eg, hematuria), and misspellings. However, few occurred in the random sample that was checked for this. Second, some errors are inevitable when classifying text extracts; however, the addition of a manual review of text strings containing words that indicate uncertainty will have minimised these. Finally, the thoroughness of the CPRD's search methods was not documented but, given their emphasis on data quality, we have no concerns about this.^2^ ^16^ Indeed, if there were any shortcomings in the CPRD's search methods, they would be unlikely to explain the differential loss of information in the hidden text between cases and controls observed in this study.

Another limitation of the study is that it did not identify whether the detection bias was caused by the recording behaviour of a small number of GP practices. However, it was important to include data from all contributing practices in order to obtain a measure of bias in CPRD studies overall. This is because the CPRD does not assess or provide quality measures about the quantity of data recorded in hidden text. Indeed, now that text data are no longer collected (see the Introduction section), the CPRD is unable to provide this information for researchers.

### Comparison with existing literature

Other than our previously published work that identified text records of non-visible haematuria,^4^ there are no directly comparable studies. Comparisons of numbers of patients overlooked because their records were made in hidden text are complicated by variations in the methods that other researchers used to identify coded records of symptoms. The more comprehensive the code list used to identify instances of symptoms, the greater the number of records identified, and the smaller the amount of data ‘lost’ in hidden text.

Ford *et al* estimated loss of clinical information about rheumatoid arthritis in CPRD hidden text. They identified text-only recording of symptoms of arthritis; for example, codes for synovitis were recorded for 179 of 6376 (2.8%) patients, but a search of the text suggested that this symptom occurred in 1168 (18.3%) patients.^17^ Their study was limited by the omission to identify if the text record was ‘positive’ (ie, reporting a current symptom) or ‘negative’ (ie, referring to an episode in the past, or confirming the absence of a symptom); therefore, the extent of data lost to analysis is likely to be overestimated. Koeling *et al*^18^ reported, in conference proceedings, that adding hidden text records increased the percentage of ovarian cancer patients with abdominal pain by a factor of 1.4, from 43% to 60%. Our findings for abdominal pain were similar, increasing by factors of 1.4 (from 419/4915 to 600/4915) and 1.3 (from 1392/3635 to 1789/3635) in bladder and pancreatic cancer cases, respectively.

Hayward *et al*^19^ examined the Consultations in Primary Care Archive data base (different to the CPRD, but also with two-tier recording) for breathlessness and wheeze before a diagnosis of ischaemic heart disease, chronic obstructive pulmonary disease or asthma. Adding hidden text records increased the percentage of patients with recorded breathlessness or wheeze from 30% to 62% in cases and from 6% to 25% in controls. This suggests a strong association between recording style and patient case–control status (χ^2^ test, p<0.001: our estimate), although this was not directly reported. This result mirrors our findings, and demonstrates that bias arising from recording method is not unique to the CPRD.

### Implications of the findings

The main implication is methodological. Our results will help researchers to assess whether their studies are likely to be vulnerable to detection bias associated with use of hidden text to record symptoms in CPRD studies. Researchers are advised that recording methods may artificially inflate risk estimates for alarm symptoms in their associated diseases, whereas it is not likely to alter risk estimates for non-alarm symptoms.

The bias demonstrated in this paper only affects high-risk features. This may not detract from the use of the risk estimates produced by cancer diagnostic studies,^5^ ^6^ as these features already generally invoke action. Indeed, the ‘corrected’ PPVs still lie near, or exceed, the 3% threshold prompting referral for suspected cancer in the latest UK national guidelines.^20^ Importantly, ‘low-risk but not no-risk’ abdominal pain and non-visible haematuria were subject to little or no bias, as shown in this and our previous study.^4^ Therefore, their risk estimates are unaltered, and they still meet criteria for inclusion in UK cancer referral guidance, justifying their recent addition.^20^ This is particularly reassuring, because low-risk symptoms are common and errors in their risk estimation would have had considerable consequences for the validity of such estimates. Nonetheless, there is a danger that the inflated risk estimates for alarm features will tend to marginalise the significance of low-risk features of cancer. However, it is rare for a low-risk feature to be included in isolation in recommendations for cancer investigation, which typically include the risk of cancer in patients presenting with two symptoms concurrently.^20^ Therefore, it is unlikely that detection bias due to loss of records in the hidden text will result in the erroneous omission of a feature from cancer recommendations.

Finally, this paper has focused on cancer, in part because it is one of the main areas where CPRD symptomatic research has concentrated. However, it is reasonable to conclude that our findings may extend to other research areas. There is the strong possibility of underestimation of symptom frequency possibly coupled with differential recording between comparison groups in all CPRD symptom studies. This does not imply that CPRD symptom-based studies are suspect, but more that they need to be interpreted carefully.

## References

[1] Williams T, van Staa T, Puri S Recent advances in the utility and use of the general practice research database as an example of a UK primary care data resource. Ther Adv Drug Saf 2012;3:89–99. 10.1177/2042098611435911 25083228 PMC4110844

[2] Herrett E, Gallagher AM, Bhaskaran K Data resource profile. Clinical Practice Research Datalink (CPRD). Int J Epidemiol 2015;44:827–36. 10.1093/ije/dyv098 26050254 PMC4521131

[3] Delgado-Rodríguez M, Llorca J Bias. J Epidemiol Community Health 2004;58:635–41. 10.1136/jech.2003.008466 15252064 PMC1732856

[4] Price SJ, Shephard EA, Stapley SA Non-visible versus visible haematuria and bladder cancer risk: a study of electronic records in primary care. Br J Gen Pract 2014;64:e584–9. 10.3399/bjgp14X681409 25179073 PMC4141616

[5] Shephard EA, Stapley S, Neal RD Clinical features of bladder cancer in primary care. Br J Gen Pract 2012;62:e598–604. 10.3399/bjgp12X654560 22947580 PMC3426598

[6] Stapley S, Peters TJ, Neal RD The risk of pancreatic cancer in symptomatic patients in primary care: a large case-control study using electronic records. Br J Cancer 2012;106:1940–4. 10.1038/bjc.2012.190 22617126 PMC3388562

[7] Shephard E, Neal R, Rose P Clinical features of kidney cancer in primary care: a case-control study using primary care records. Br J Gen Pract 2013;63:e250–5. 10.3399/bjgp13X665215 23540481 PMC3609472

[8] Shephard EA, Neal RD, Rose P Quantifying the risk of multiple myeloma from symptoms reported in primary care patients: a large case-control study using electronic records. Br J Gen Pract 2015;65:e106–13. 10.3399/bjgp15X683545 25624306 PMC4325456

[9] Shephard EA, Neal RD, Rose PW Quantifying the risk of Hodgkin's lymphoma in symptomatic primary care patients aged ≥40 years: a case-control study using electronic records. Br J Gen Pract 2015;65:e289–94. 10.3399/bjgp15X684805 25918333 PMC4408504

[10] Shephard EA, Neal RD, Rose PW Quantifying the risk of non-Hodgkin's lymphoma in symptomatic primary care patients aged ≥40 years: a large case-control study using electronic records. Br J Gen Pract 2015;65:e281–8. 10.3399/bjgp15X684793 25918332 PMC4408518

[11] Stapley S, Peters TJ, Neal RD The risk of oesophago-gastric cancer in symptomatic patients in primary care: a large case-control study using electronic records. Br J Cancer 2013;108:25–31. 10.1038/bjc.2012.551 23257895 PMC3553533

[12] Walker S, Hyde C, Hamilton W Risk of uterine cancer in symptomatic women in primary care: case-control study using electronic records. Br J Gen Pract 2013;63:e643–8. 10.3399/bjgp13X671632 23998845 PMC3750804

[13] Walker S, Hyde C, Hamilton W Risk of breast cancer in symptomatic women in primary care: a case-control study using electronic records. Br J Gen Pract 2014;64:e788–93. 10.3399/bjgp14X682873 25452544 PMC4240152

[14] Rutjes AW, Reitsma JB, Coomarasamy A Evaluation of diagnostic tests when there is no gold standard. A review of methods. Health Technol Assess 2007;11:iii, ix-51. 10.3310/hta11500 18021577

[15] Holtedahl KA A method of calculating diagnostic indexes for possible cancer symptoms in general practice. Allgemein Medizin 1990;19:74–9.

[16] The General Practice Research Database. GPRD recording guidelines for Vision users. London: Crown Publishing, 2004.

[17] Ford E, Nicholson A, Koeling R Optimising the use of electronic health records to estimate the incidence of rheumatoid arthritis in primary care: what information is hidden in free text? BMC Med Res Methodol 2013;13:105 10.1186/1471-2288-13-105 23964710 PMC3765394

[18] Koeling R, Tate AR, Carroll JA Automatically estimating the incidence of symptoms recorded in GP free text notes. In: First international workshop on managing interoperability and complexity in health systems. Glasgow, UK, 2011.

[19] Hayward RA, Chen Y, Croft P Presentation of respiratory symptoms prior to diagnosis in general practice: a case-control study examining free text and morbidity codes. BMJ Open 2015;5:e007355 10.1136/bmjopen-2014-007355 PMC4466603 26070795

[20] National Institute for Health and Care Excellence. Suspected cancer: recognition and referral [NG12]. London: NICE, 2015. 26180880

