# Supplementary material for: Is omission of free text records a possible source of data loss and bias in Clinical Practice Research Datalink studies? A case–control study
Source: BMJ Open. 2016 May 13;6(5):e011664. doi: 10.1136/bmjopen-2016-011664 (PMC4874123; doi:10.1136/bmjopen-2016-011664)
Supplement: Supplementary appendix 1 [file bmjopen-2016-011664supp_appendix1.pdf]

Are free text records a possible source of detection bias in Clinical Practice Research Datalink studies? A case–control study

Supplementary appendix 1 – full inclusion and exclusion criteria

Recruitment period

The baseline population consisted of all acceptable patients registered with CPRD up-to-standard practices in the study period, which was 1 January 2000 to 31 December 2009, inclusive.

Inclusion criteria for bladder and pancreatic cancer cases

Inclusion criteria for cases consisted of:

- Being registered with a CPRD up-to-standard practice during the study period
- Having a clinical or referral record in the electronic medical record of incident bladder or pancreatic cancer in the study period while they were also registered with an up-to-standard CPRD practice (code lists are available in Supplementary appendix 2)
- Being aged ≥40 years at diagnosis
- Being registered at the CPRD practice for at least 1 year before diagnosis
- Having gender clearly recorded as either ‘male’ or ‘female’

If more than 5,000 patients met these criteria, a random sample of 5,000 was taken. This affected bladder cancer, but not pancreatic cancer.

Are free text records a possible source of detection bias in Clinical Practice Research Datalink studies? A case–control study

Exclusion criteria for bladder and pancreatic cancer cases

Exclusion criteria for cases consisted of:

- Having a secondary cancer
- Being diagnosed with any cancer before 1 January, 2000
- Not consulting in primary care in the year before the cancer diagnosis
- Not having any matched controls

Inclusion criteria for bladder and pancreatic cancer controls

Controls were selected from the same baseline population that yielded the cases. Up to five controls were selected per case, as this increased the power of the study.[1] Cases and controls were matched on sex, age and GP practice using observation window matching. Inclusion criteria for controls consisted of:

- Being registered with the same GP practice as their matched case when the latter was diagnosed with cancer
- Being aged ≥40 years when their case was diagnosed with cancer
- Having at least one entry in the electronic medical record in the year prior to cancer diagnosis in the case
- Being alive on the date on which their case was diagnosed with cancer

Are free text records a possible source of detection bias in Clinical Practice Research Datalink studies? A case–control study

Exclusion criteria for bladder and pancreatic cancer controls

Exclusion criteria for controls consisted of:

- Exclusion of their matched case for any reason
- Being diagnosed with the same cancer as their matched case before 1 January, 2000
- Being diagnosed with the same cancer as their matched case after 1 January, 2000 – as this would mean they were eligible to be a case themselves
- Being a control for another case

Bladder cancer study cases

In total, 4,935 potential cases were identified as having a clinical or referral record of incident bladder cancer, as defined by the list of Read codes agreed between the primary investigator (Professor Hamilton) and the CPRD research team (see Supplementary Appendix 2).

Excluded cases

After application of the exclusion criteria, 20 potential cases (*n* = 19 men, *n* = 1 woman) were excluded from the study, for reasons given in Table 1. There were no cases who had not consulted their GP in the year prior to their diagnosis, leaving a total of 4,915 cases in the study.

Are free text records a possible source of detection bias in Clinical Practice Research Datalink studies? A case–control study

Bladder cancer study controls

In total, 24,098 patients were identified by the CPRD as eligible for consideration as controls.

Excluded controls

After application of the exclusion criteria, 2,380 potential controls (*n* = 1,958 men, *n* = 422 women) were excluded from the study for reasons given in Table 1.

Table 1 Bladder cancer study exclusions

Case or control	Reason for exclusion	Number of patients excluded
**Case**	No matched control identified	13
	Metastatic cancer present	7
	Subtotal	*20*
**Control**	Diagnosed with bladder cancer after the year 2000	125
	Diagnosed with bladder cancer before the year 2000	134
	No data recorded in their medical record in the analysis period	2,086
	Matched to a case who was excluded because they had metastatic cancer	35
	Subtotal	2,380
**Total**		**2,400**

Are free text records a possible source of detection bias in Clinical Practice Research Datalink studies? A case–control study

Bladder cancer study matching

After exclusions, there were 4,915 bladder cancer cases matched to 21,718 controls on age, sex and GP practice, as reported in Table 2. The majority of cases were matched to at least four controls.

Table 2 Bladder cancer study matching

No. (%) of cases with:					
**1 control**	**2 controls**	**3 controls**	**4 controls**	**5 controls**	**Total**
57 (1.2)	160 (3.3)	419 (8.5)	1,311 (26.7)	2,968 (60.4)	4,915

Pancreatic cancer study cases

In total, 3,647 potential cases were identified as having a clinical or referral record of incident pancreatic cancer, as defined by the list of Read codes agreed between the primary investigator (Professor Hamilton) and the CPRD (see Supplementary Appendix 2).

Excluded cases

After application of the exclusion criteria, 12 potential cases were excluded from the study, for reasons given in Table 3. There were no cases who had not consulted their GP in the year prior to their diagnosis, leaving a total of 3,635 cases in the study.

Are free text records a possible source of detection bias in Clinical Practice Research Datalink studies? A case–control study

Pancreatic cancer study controls

In total, 17,977 patients were identified by the CPRD as eligible for consideration as controls.

Excluded controls

After application of the exclusion criteria, 1,518 potential controls were excluded from the study for reasons given in Table 3.

Table 3 Pancreatic cancer study exclusions

Case or control	Reason for exclusion	Number of patients excluded
**Case**	No matched control identified	2
	Tumour not originating in the pancreas	10
	Subtotal	*12*
**Control**	Diagnosis of pancreatic cancer	64
	No data in the year before diagnosis	1,414
	Case excluded	40
	Subtotal	*1,518*
**Total**		**1,530**

Are free text records a possible source of detection bias in Clinical Practice Research Datalink studies? A case–control study

Pancreatic cancer study matching

After exclusions, there were 3,635 cases matched to 16,459 controls on age, sex and GP practice, as reported in Table 4.

Table 4 Pancreatic cancer study matching

No. of cases with:					
**1 control**	**2 controls**	**3 controls**	**4 controls**	**5 controls**	**Total**
26 (0.7)	90 (2.5)	251 (6.9)	840 (23.1)	2,428 (66.8)	3,635

Reference

1. Breslow NE, Day NE. *Statistical Methods in Cancer Research. Volume I. The Analysis of Case-control Studies*. Geneva: IARC Scientific Publications, no. 32, 1980, pp. 5-338.
