# Supplementary material for: Is omission of free text records a possible source of data loss and bias in Clinical Practice Research Datalink studies? A case–control study
Source: BMJ Open. 2016 May 13;6(5):e011664. doi: 10.1136/bmjopen-2016-011664 (PMC4874123; doi:10.1136/bmjopen-2016-011664)
Supplement: Supplementary appendix 2 [file bmjopen-2016-011664supp_appendix2.pdf]

Are free text records a possible source of detection bias in Clinical Practice Research Datalink studies? A case–control study

Supplementary Appendix 2 – code lists

Bladder cancer

Description	Read code
Malignant neoplasm of urinary bladder	B49..00
[M]Transitional cell carcinoma NOS	BB43.00
Carcinoma *in situ* of bladder	B837.00
[M]Papillary transitional cell carcinoma	BB4A.00
Neoplasm of unspecified nature of bladder	BA04.00
Malignant neoplasm of anterior wall of urinary bladder	B493.00
[M]Transitional cell carcinoma *in situ*	BB42.00
Malignant neoplasm of ureteric orifice	B496.00
Malignant neoplasm of urinary bladder NOS	B49z.00
Malignant neoplasm of lateral wall of urinary bladder	B492.00
Malignant neoplasm of other site of urinary bladder	B49y.00
Malignant neoplasm of trigone of urinary bladder	B490.00
Malignant neoplasm of bladder neck	B495.00
Malignant neoplasm of posterior wall of urinary bladder	B494.00
Malignant neoplasm of urachus	B497.00
Malignant neoplasm of dome of urinary bladder	B491.00
Malignant neoplasm, overlapping lesion of bladder	B49y000
[M]Transitional cell carcinoma, spindle cell type	BB47.00

Are free text records a possible source of detection bias in Clinical Practice Research Datalink studies? A case–control study

Pancreatic cancer codes

Description	Read code
Malignant neoplasm of pancreas	B17..00
Malignant neoplasm of head of pancreas	B170.00
[M]Insulinoma NOS	BB5B200
Malignant neoplasm of ampulla of Vater	B162.00
Carcinoma *in situ* of pancreas	B80z000
Carcinoma *in situ* of ampulla of Vater	B808600
[M]Gastrinoma and carcinomas	BB5C.00
[M]Glucagonoma, malignant	BB5B500
Malignant neoplasm of pancreas NOS	B17z.00
Malignant neoplasm of pancreatic duct	B173.00
[M]Gastrinoma NOS	BB5C000
Malignant neoplasm of Islets of Langerhans	B174.00
Malignant neoplasm of tail of pancreas	B172.00
Malignant neoplasm of body of pancreas	B171.00
[M]Gastrinoma or carcinoma NOS	BB5Cz00
Malignant neoplasm of other specified sites of pancreas	B17y.00
[M]Gastrinoma, malignant	BB5C100
Endocrine tumour of pancreas	B717011
[M]Glucagonoma NOS	BB5B400
[M]Islet cell carcinoma	BB5B100
[M]Insulinoma, malignant	BB5B300

Are free text records a possible source of detection bias in Clinical Practice Research Datalink studies? A case–control study

Description	Read code
Malignant neoplasm of specified site of pancreas NOS	B17yz00
Malignant neoplasm of ectopic pancreatic tissue	B17y000
Malignant neoplasm, overlapping lesion of pancreas	B175.00
[M]Mixed islet cell and exocrine adenocarcinoma	BB5B600

Abdominal pain codes

Description	Read code
Abdominal migraine	F262200
Abdominal migraine - symptom	1967.00
Abdominal pain	1969.00
Abdominal pain type	196..11
Abdominal wall pain	1969000
Angina - abdominal	J421.11
Appendicular colic	J23z000
Biliary colic	1965.00
Biliary colic	J642200
Biliary colic symptom	1965.11
Central abdominal pain	1971.00
Colicky abdominal pain	1962.00
Epigastric pain	1972.00
General abdominal pain-symptom	197A.11

Are free text records a possible source of detection bias in Clinical Practice Research Datalink studies? A case–control study

Description	Read code
Generalised abdominal pain	197A.00
Griping pain	1DC5.00
Left iliac fossa pain	1978.00
Lower abdominal pain	197C.00
Non-colicky abdominal pain	1963.00
O/E - abd. pain - L.iliac	25CA.00
O/E - abd. pain - L.lumbar	25C7.00
O/E - abd. pain - R. iliac	25C8.00
O/E - abd. pain - R.lumbar	25C5.00
O/E - abd. pain - epigastrium	25C3.00
O/E - abd. pain - hypogastrium	25C9.00
O/E - abd. pain - umbilical	25C6.00
O/E - abd.pain-L.hypochondrium	25C4.00
O/E - abd.pain-R.hypochondrium	25C2.00
O/E - abdo. pain on palpation	25C..00
O/E - epigastric pain on palp.	25C..11
O/E - guarding - epigastrium	25D3.00
O/E - guarding - hypogastrium	25D9.00
O/E - guarding - umbilical	25D6.00
O/E - guarding of abdomen	25D..11
O/E - guarding-L.hypochondrium	25D4.00
O/E - guarding-R.hypochondrium	25D2.00

Are free text records a possible source of detection bias in Clinical Practice Research Datalink studies? A case–control study

Description	Read code
O/E - rebound - epigastrium	25E3.00
O/E - rebound tenderness	25E..00
O/E - rebound tenderness NOS	25EZ.00
O/E - rebound-R.hypochondrium	25E2.00
O/E - umbilical pain on palp.	25C..14
O/E -abd.pain on palpation NOS	25CZ.00
Right iliac fossa pain	1977.00
Right upper quadrant pain	197D.00
Shoulder pain from abdomen	1964.00
Site of GIT pain	197..00
Site of GIT pain NOS	197Z.00
Site of abdominal pain	197..13
Subcostal pain	197..14
Suprapubic pain	1979.00
Type of GIT pain	196..00
Type of GIT pain - symptom	196..12
Type of GIT pain NOS	196Z.00
Upper abdominal pain	197B.00
[D]Abdominal colic	R090100
[D]Abdominal cramps	R090400
[D]Abdominal migraine	R090D00
[D]Abdominal pain	R090.00

Are free text records a possible source of detection bias in Clinical Practice Research Datalink studies? A case–control study

Description	Read code
[D]Abdominal pain NOS	R090z00
[D]Acute abdomen	R096.00
[D]Colic NOS	R090200
[D]Epigastric pain	R090500
[D]Gas pain (abdominal)	R073200
[D]Groin pain	R090B00
[D]Hypochondrial pain	R090700
[D]Left upper quadrant pain	R090K00
[D]Nonspecific abdominal pain	R090N00
[D]Other specified abdominal pain	R090y00
[D]Pain in left iliac fossa	R090A00
[D]Pain in right iliac fossa	R090900
[D]Recurrent acute abdominal pain	R090E00
[D]Renal colic, unspecified	R080000
[D]Right upper quadrant pain	R090J00
[D]Suprapubic pain	R090800
[D]Umbilical pain	R090600
[D]Upper abdominal pain	R090H00
[X]Other and unspecified abdominal pain	Ryu1100
[X]Pain localized to other parts of lower abdomen	Ryu1000

Are free text records a possible source of detection bias in Clinical Practice Research Datalink studies? A case–control study

Haematuria

Description	Read code
Haematuria	K197.00
Haematuria - symptom	1A45.12
Blood in urine - symptom	1A45.11
Blood in urine - haematuria	1A45.00
Clot haematuria	K197400
Recurrent benign haematuria syndrome	K032100
Frank haematuria	K197300
Painless haematuria	K197000
RBCs- red blood cells in urine	46G2.11
Urine: trace non-haemol. blood	4693.00
Urine blood test = +++	4697.00
Urine: trace haemolysed blood	4694.00
Urine blood test = +	4695.00
Recurrent and persistent haematuria	K0A2.00
Traumatic haematuria	K197.11
Urine blood test = ++	4696.00
Painful haematuria	K197100
Urine microscopy:RBC's present	46G2.00
Urine: red - blood	4625.00
Recurrent and persistent haematuria, dense deposit disease	K0A2600
Essential haematuria	K197.12

Description	Read code
Recur+persist haematuria difus crescentic glomerulonephritis	K0A2700
Recur+persist haematuria difus membranous glomerulonephritis	K0A2200
Recur+persist haematuria, focal+segmental glomerular lesions	K0A2100
Recurrent+persistnt haematuria minor glomerular abnormality	K0A2000

Jaundice

Description	Read code
[D]Jaundice (not of newborn)	R024.00
[D]Jaundice	R024111
Obstructive jaundice NOS	J66y600
O/E - jaundiced	2274.11
Jaundice - symptom	1675.11
Yellow - symptom	1675.12
[D]Icterus NOS	R024100
Yellow/jaundiced colour	1675.00
O/E - jaundiced colour	2274.00
[D]Jaundice (not of newborn) NOS	R024z00
