# Supplementary material for: Is omission of free text records a possible source of data loss and bias in Clinical Practice Research Datalink studies? A case–control study
Source: BMJ Open. 2016 May 13;6(5):e011664. doi: 10.1136/bmjopen-2016-011664 (PMC4874123; doi:10.1136/bmjopen-2016-011664)
Supplement: Supplementary appendix 3 [file bmjopen-2016-011664supp_appendix3.pdf]

Are free text records a possible source of detection bias in Clinical Practice Research Datalink studies? A case–control study

Supplementary Appendix 3 – Gold standard construction and text extract classification

Introduction

The Clinical Practice Research Datalink (CPRD) searched the text fields of the electronic medical records of all participants in the bladder (n=26,633 patients) and pancreatic (n=20,094 patients) cancer studies for key words relating to haematuria, jaundice or abdominal pain. This identified 13,853 text records, which were in string variable format and needed to be converted into binary variables suitable for analysis – symptom present or symptom absent. To do this, we devised a classification procedure that included a 'triage' algorithm run in Stata (version 13), followed by manual review of selected text extracts. This enabled the consistent application of rules governing the classification of text extracts and their efficient conversion to binary variables.

The 'semi-automated' classification process was validated by comparison of its performance with that of a gold standard. Its performance characteristics are reported in terms of sensitivity, specificity, and positive and negative predictive values, as well as the weighted kappa statistic.[1,2]

Gold standard construction

The gold standard was created in four stages: an initial one in which two practising GPs independently classified a random sample of 100 text strings (the pilot study); a second

stage where the GPs clarified the criteria underpinning their decisions; and a third step in which they re-assessed the same sample, applying the agreed criteria.[1] In the final stage, text strings were dropped if the GPs could not agree as to their meaning, or agreed that the extract was wholly uninterpretable. This left a gold standard of text extracts whose classification was confirmed and agreed.

Stage 1: the pilot study

The results of the first independent assessment of the random sample of 100 text strings carried out by the two raters in the pilot study are displayed in Table 1**Error! Reference source not found.**.

The marginal totals for the three categories were symmetrically unbalanced: for both observers the prevalence of 'current' haematuria' (74% for rater 1 and 77% for rater 2) greatly exceeded that of either 'no current haematuria' (12% for rater 1 and 15% for rater 2) or 'uncertain' (i.e. meaning unclear) (14% for rater 1 and 8% for rater 2).

Table 1 Results of independent assessments of the random sample of 100 text strings

		Rater 1			Total
		**Current haematuria**	**Uncertain**	**No current haematuria**	
**Rater 2**	**Current haematuria**	**68**	7	2	77
	**Uncertain**	4	**2**	2	8
	**No current haematuria**	2	5	**8**	15

	Rater 1			Total
	Current haematuria	Uncertain	No current haematuria	
**Total**	74	14	12	100

The observed proportion of agreement between the raters was (68 + 2 + 8)/100 (78%); however, this measure overestimates the true level of agreement as some of this will have arisen purely by chance. The chance-corrected weighted kappa was 0.7 (95% CI: 0.6–0.9). In terms of inter-rater agreement, this is interpreted as fair to good by Landis and Koch,[3] as good by Altman[4] and as substantial by McGinn *et al.*[5] The symmetrical imbalance within the sample in favour of 'current haematuria' means that the reported chance-corrected weighted kappa is likely to underestimate the level of agreement.[2]

Overall, raters 1 and 2 agreed that 2% of the observations were wholly uninterpretable.

Stage 2: clarification of category definitions

The category definitions were agreed as follows:

'**Symptom negative**' – used to describe those observations in which symptoms were explicitly or implicitly described as absent at the time the patient consulted the GP.

'**Symptom positive**' – used to describe those observations in which symptoms were contemporary.

‘**Uncertain**’ – used to describe when the patient’s status regarding the symptom could not be ascertained with certainty.

Stage 3: results after re-assessment following agreement of category definitions

After discussion and agreement of the definitions for the three categories, the two raters re-assessed the same random set of observations. The results are reported in Table 2. The anticipation was that fewer observations would be classed as ‘uncertain’. Rater 1 did tend to assign this category less frequently, with its percentage reducing from 14% to 3%. However, the use of this category by rater 2 remained fairly steady at 8% and then 11%.

Table 2 Results of independent re-assessment of the random sample of 100 text strings

		Rater 1 (revised)			Total
		Current haematuria	Uncertain	No current haematuria	
Rater 2 (revised)	Current haematuria	**78**	1	4	83
	Uncertain	2	**2**	7	11
	No current haematuria	0	0	**6**	6
Total		80	3	17	100

The re-assessment did not seem to improve the overall level of agreement, which remained similar to that in the pilot study. While the observed proportion of agreement rose to 86%, the chance-corrected weighted kappa remained similar to the value obtained previously, at 0.7 (95% CI: 0.5–0.9). The marginal totals for the three categories remained symmetrically unbalanced with the category ‘current haematuria’ dominant for both raters. Therefore, the weighted kappa still tended to underestimate the true level of agreement.[2]

After clarifying the definitions of each category, raters 1 and 2 could not agree as to the classification of 14 observations, but agreed that 2 observations were unclear.

Stage 4: Finalising the gold standard

The uninterpretable extracts (n=2) and extracts whose meaning the raters could not agree (n=14) were dropped. Therefore, the gold standard consisted of 84 extracts whose meaning was fully agreed by the raters.

Performance of the final classification against the gold standard

The semi-automated classification procedure was used to classify the same extracts used to create the gold standard. The output was compared with that of the gold standard in a contingency table (see

Table 3). As the gold standard provides error-free classification, all false-positives and false-negatives can be attributed with certainty to error in the classification procedure.

Table 3 Two-way tabulation of the semi-automated classification procedure's output against that of the reference standard

		Gold standard		Total
		**Current haematuria**	**No current haematuria**	
**Final classification output**	Current haematuria	75	0	75
	Uncertain	3	0	3
	No current haematuria	0	6	6
**Total**		78	6	84

The observed proportion of agreement between the classification system and the reference standard was (75+6)/84 = 96%. The chance-corrected weighted kappa was 0.9 (standard error = 0.1).

Sensitivity analyses

Sensitivity analyses were carried out as illustrated by the purple and black lines in

Are free text records a possible source of detection bias in Clinical Practice Research Datalink studies? A case–control study

Table 3. The category ‘Uncertain’ in the final classification output was merged first with ‘Current haematuria’ and then with ‘No current haematuria’ to derive the following binary systems:

1. ‘Current haematuria’ or “Not ‘Current haematuria’” (i.e. merged groups ‘No current haematuria’ and ‘Uncertain’). The black lines in

Are free text records a possible source of detection bias in Clinical Practice Research Datalink studies? A case–control study

2. Table 3 above illustrate this.

3. ‘No current haematuria’ or “Not ‘No current haematuria’” (i.e. merged groups ‘Current haematuria’ and ‘Uncertain’). The purple lines in

4. Table 3 above illustrate this.

Two-way tabulation of the semi-automated classification procedure's output against that of the gold standard is reported in Table 4, under sensitivity analysis 1.

Table 4 Two-way tabulation of the semi-automated classification procedure's output against that of the reference standard under sensitivity analysis 1

		Reference standard		Totals
		**Current haematuria**	**No current haematuria**	
**Final classification output**	**Current haematuria**	75	0	75
	**Not 'Current haematuria'**	3	6	9
**Totals**		78	6	84

In this analysis, the sensitivity is 75/78 (96%), the specificity and positive predictive value (PPV) are both 100%. The negative predictive value (NPV) is 6/9 (67%). The weighted kappa score is 0.8 (standard error = 0.1).

Two-way tabulation of the semi-automated classification procedure's output against that of the reference standard is reported in Table 5, under sensitivity analysis 2.

Under this analysis, sensitivity, specificity, PPV and NPV were all 100%, and the weighted kappa score is 1.0 (standard error = 0.1).

Table 5 Two-way tabulation of the semi-automated classification procedure's output against that of the reference standard under sensitivity analysis 2

		Reference standard		Totals
		**Current haematuria**	**Not 'Current haematuria'**	
**Final classification output**	**Not 'No current haematuria'**	78	0	78
	**No current haematuria**	0	6	6
**Totals**		78	6	84

Classification of text extracts

The results of text classification are reported below, for the bladder (

Table 6) and pancreatic (

Table 7) cancer datasets separately.

Table 6 Text extract classification for visible haematuria, abdominal pain and jaundice in the bladder cancer dataset

Stage of classification		No. of observations in the bladder cancer dataset relating to:		
		Visible haematuria	Abdominal pain	Jaundice
**By algorithm**	No current symptom	370	341	95
	Unclear	521	252	60
	Current symptom	3,780	1,356	149
	Total	*4,671*	*1,949*	*304*
**After manual check**	No current symptom – drop	774	538	178
	Unclear – drop	58	17	8
	**Current symptom**	**3,839**	**1,394**	**118**
**Final classification**	Dropped: the symptom was also recorded as a Read code	972	271	21
	Dropped: paired medcode unidentifiable	2	0	0
	Dropped: duplicated record	166	41	9
	**Retained: true text-only record**	**2,699**	**1,082**	**88**

Table 7 Text extract classification for jaundice, abdominal pain and visible haematuria in the pancreatic cancer dataset

Stage of classification		No. of observations in the pancreatic cancer dataset relating to:		
		Jaundice	Abdominal pain	Visible haematuria
**By algorithm**	No current symptom	285	395	77
	Unclear	139	371	101
	**Current symptom**	**2,391**	**2,852**	**318**
	Total	*2,815*	*3,618*	*496*
**After manual verification**	No current symptom	432	573	156
	Unclear	140	66	32
	**Current symptom**	**2,243**	**2,979**	**308**
**Final classification**	Dropped: the symptom was also recorded as a Read code	471	687	49
	Dropped: paired medcode unidentifiable	0	0	0
	Dropped: duplicated record	133	100	8
	**Retained: true text-only record**	**1,639**	**2,192**	**251**

References

1. Rutjes AW, Reitsma JB, Coomarasamy A, Khan KS, Bossuyt PM. Evaluation of diagnostic tests when there is no gold standard. A review of methods. *Health Technol Assess* 2007;**11**(50):iii, ix-51

2. Feinstein AR, Cicchetti DV. High agreement but low kappa: I. The problems of two paradoxes. *J Clin Epidemiol* 1990;**43**(6):543-9.

3. Landis J, Koch G. The measurement of interobserver agreement for categorical data. *Biometrics* 1977;**33**:159-74.

4. Altman DG. *Practical Statistics for Medical Research.* London: Chapman & Hall, 1991.

5. McGinn T, Wyer PC, Newman TB, Keitz S, Leipzig R, For GG. Tips for learners of evidence-based medicine: 3. Measures of observer variability (kappa statistic). *CMAJ* 2004;**171**(11):1369-73.
